# Detection of Autonomic Dysfunction in Hemodialysis Patients Using the Exercise Treadmill Test: The Role of the Chronotropic Index, Heart Rate Recovery, and R-R Variability

**DOI:** 10.1371/journal.pone.0128123

**Published:** 2015-06-04

**Authors:** Maria Angela M. Q. Carreira, André B. Nogueira, Felipe M. Pena, Marcio G. Kiuchi, Ronaldo C. Rodrigues, Rodrigo R. Rodrigues, Jorge P. S. Matos, Jocemir R. Lugon

**Affiliations:** 1 Department of Medicine, Cardiology Division, Medical School, Universidade Federal Fluminense, Rio de Janeiro, Brazil; 2 Department of Medicine, Nephrology Division, Medical School, Universidade Federal Fluminense, Rio de Janeiro, Brazil; The University of Manchester, UNITED KINGDOM

## Abstract

Autonomic dysfunction is highly prevalent in hemodialysis patients and has been implicated in their increased risk of cardiovascular mortality.

**Objective:**

To evaluate the ability of different parameters of exercise treadmill test to detect autonomic dysfunction in hemodialysis patients.

**Methods:**

Cross-sectional study involving hemodialysis patients and a control group. Clinical examination, blood sampling, echocardiogram, 24-hour Holter, and exercise treadmill test were performed. A ramp treadmill protocol symptom-limited with active recovery was employed.

**Results:**

Forty-one hemodialysis patients and 41 controls concluded the study. There was significant difference between hemodialysis patients and controls in autonomic function parameters in 24h-Holter and exercise treadmill test. Probability of having autonomic dysfunction in hemodialysis patients compared to controls was 29.7 at the exercise treadmill test and 13.0 in the 24-hour Holter. Chronotropic index, heart rate recovery at the 1st min, and SDNN at exercise were used to develop an autonomic dysfunction score to grade autonomic dysfunction, in which, 83% of hemodialysis patients reached a scoring ≥2 in contrast to 20% of controls. Hemodialysis was independently associated with either altered chronotropic index or autonomic dysfunction scoring ≥2 in every tested model (OR=50.1, P=0.003; and OR=270.9, P=0.002, respectively, model 5).

**Conclusion:**

The exercise treadmill test was feasible and useful to diagnose of the autonomic dysfunction in hemodialysis patients. Chronotropic index and autonomic dysfunction scoring ≥2 were the most effective parameters to differentiate between hemodialysis patients and controls suggesting that these variables portrays the best ability to detect autonomic dysfunction in this setting.

## Introduction

Cardiovascular morbidity and mortality remain exceptionally high in maintenance hemodialysis (HD) patients. Autonomic dysfunction (AD) is highly prevalent in end-stage renal disease patients undergoing HD treatment [1] and has been implicated in the increased risk of sudden death in this population [2]. Studies in dialysis patients have consistently demonstrated a poor autonomic function which is associated with increased mortality rate [3,4]. The 24h-Holter is the most frequent method to evaluated AD. The exercise treadmill test (ETT) has mainly been used to evaluate cardiorespiratory fitness in this population but it also allows measurement of changes in heart rate (HR) under physical stress and can be used to assess autonomic function [5–8]. On the other side, HD patients have high levels of inflammatory markers such as C-reactive protein, which have also been associated with autonomic dysfunction and increased risk of mortality [8,9].

The aim of this study was to evaluate the ability of different parameters of ETT to detect AD in maintenance HD patients.

## Methods

### Study population

We conducted a cross-sectional study with end stage renal disease patients on HD 3 times a week (4-hour duration sessions), for at least 3 months and a control group matched by gender and age without overt kidney disease. Hemodialysis patients were recruited from a single dialysis center and the control group consisted of individuals referred for exercise testing at the University Hospital. Written informed consent was obtained and approved, as well the protocol of study, by the ethical committee of the medical school (Comitê de Ética em Pesquisa da Faculdade de Medicina da Universidade Federal Fluminense—Hospital Universitário Antonio Pedro, n°: CAAE: 0125.0258.000–10). Chronic medications, including those for blood pressure control, were not discontinued during the study.

Exclusion criteria were as follow: impaired gait that prevented walking on the treadmill, arrhythmias preventing proper assessment of heart rate, and the presence of symptomatic heart disease. Cardiac evaluation was always accomplished in a middle of week in non-dialytic day and consisted of: clinical examination, transthoracic echocardiogram, 24-h Holter, and ETT in the interdialytic period.

### Echocardiography

A two-dimensional transthoracic echocardiography was performed with GE VIVID 7 System (General Electric Company, USA) to assess left ventricle wall motion and systolic and diastolic ventricular function.

### 24h-Holter

Patients underwent a 24h-Holter (Galix Biomedical Instrumentation, Florida, USA). A 3-channel recorder was used to record the electrocardiographic traces. A time domain analysis of HRV was performed and the following parameters were obtained: a) SDNN, standard deviation (SD) of all normal RR intervals (NN); b) SDANN, SD of the averages of 5-min NN intervals over 24-h; c) rMSSD, the square root of the mean of the square of successive NN intervals; and d) triangular index (TI), integral of the density distribution (that is, the number of all NN intervals) divided by the maximum density distribution.

Cutoff values adopted in the present study were derived from a previous study in which SDNN <50 msec, SDANN <40 msec, rMSSD <15 msec, and TI <15 were definitely associated with increased cardiovascular mortality in HD patients [10].

### Exercise treadmill test

Patients underwent ETT on a treadmill ramp protocol using the program Ergo13 (Heart Ware Co., Minas Gerais, Brazil). The test was symptom limited, scheduled for ten minutes with active recovery for 2 minutes under 40% of the speed and incline of peak effort. An automatic electrocardiogram was recorded in 13 simultaneous leads before exercise in supine and in standing, at peak exercise, and at every minute of recovery. The following HR related to ETT parameters of AD were analyzed: chronotropic index, heart rate recovery (HRR) at 1^st^, 2^nd^, 3^rd^, 4^th^ and 5^th^min, resting HR/HRR index and HRV (SDNN and rMSSD) during exercise and at the recovery period. The predicted maximal VO_2_ in ramp protocol was reduced by 20% in HD patients. The maximal VO_2_ peak exercise was obtained by the Foster formula backed with hands. The functional aerobic impairment was calculated using the formula: maximum predicted VO_2_–peak VO_2_) / maximal predicted VO_2_] x100. The predicted maximal HR was calculating by: 208–(0.7 x age). Chronotropic index was calculated as: [peak HR-resting HR] / [(max predicted HR—resting HR]. Chronotropic incompetence was said to be present when values of chronotropic index were <0.80 (or <0.62 if in use of betablocker) [11,12]. The HRR was calculated from the absolute differences between peak HR values and the HR values in every minute of recovery. Values in HRR at 1^st^min >12 bpm and at 2^nd^min >22bpm were considered abnormal [13]. The resting HR/HRR index was calculated as (resting HR-HRR)/RHR and values above 0.72 were considered abnormal [14]. Heart rate variability in ETT was obtained by software specially designed for the study conveying electrocardiographic data findings of Ergo13 for Cardio Smart (Cardios, São Paulo, Brazil) during exercise and recovery periods separately.

### Blood analysis

Blood samples were performed 30 min before the exercise test for determination of ultrasensitive C-reactive protein in both groups and creatinine in control group (to exclude overt renal disease). C-reactive protein was obtained by a immunoturbidimetric assay (Dimension RxLMax, Siemens, Berlin, Germany).

### Statistics

Results were expressed as mean and SD for normal distribution and median and range otherwise. Categorical variables were expressed as frequencies and compared using the Chi-square test. Comparisons between two continuous variables were accomplished by the t test (for normal distribution) or its nonparametric equivalent (Mann-Whitney test). Logistic regression was used to analyze associations. P<0.05 was considered significant. Analyses were performed using SPSS for Windows version 18.0 (SPSS Inc., Chicago, IL, USA).

## Results

A total of 125 patients from a single dialysis center were initially evaluated. Nine were promptly excluded due to gait impairment. Of the 116 remaining, 59 agreed to participate and signed the consent form. Eighteen patients were excluded: 10 did not show up for the exams, 4 had a past myocardial infarction, 2 had arrhythmia, 1 had bilateral fistula, and 1 had current pulmonary infection. At the end, 41 HD patients concluded the study. The most common etiologies of the renal disease were: hypertensive nephrosclerosis (56%), chronic glomerulonephritis (17%), polycystic kidney (10%), and diabetic nephropathy (7%). The general features of patients and controls are in Table 1.

**Table 1:** General features of patients.

	HD patients	Controls	P value
Age, years	50 ± 14^a^	50 ± 13	0.975
Male, %	21 (51.2)^b^	21 (51.2)	1.000
Race, non-white %	27 (65.9)	21 (51.2)	0.391
Body mass index, kg/m^2^	25.1 ± 5.1	27.3 ± 4.1	0.030
HD vintage, months	67.2 ± 47.3	n.a.	-
Diabetes, %	4 (9.8)	5 (12.2)	0.724
Smoking, %	3 (9.1)	8 (20.5)	0.180
Familial CAD,%	15 (36.6)	17 (41.5)	0.520
Familial hypertension, %	26 (63.4)	21 (51.2)	0.525
Sedentary, %	33 (80.5)	24 (58.5)	0.031
Use of blood pressure drugs, %	33 (80.5)	20 (48.8)	0.003
Beta-blocker	14 (34.1)	7 (17.1)	0.077
Diuretic	2 (4.9)	8 (19.5)	0.043
Calcium channel blocker	5 (12.2)	2 (4.9)	0.236
ACE inhibitor/ARB	12 (29.3)	16 (39.0)	0.352
Clonidine	8 (19.5)	-	0.003
Alfa-blocker	6 (14.6)	-	0.011
C-reactive protein, mg/dL	1.02 ± 1.20	0.47 ± 0.07	0.010
Hemoglobin, g/dL	11.5±1.4	13.8±1.2	<0.001
eGFR (MDRD study), ml/min/1.73m^2^	n.a.	87.5 ± 23.1	-

ACE, angiotensin-converting—enzyme; ARB, AT1–receptor blocker; CAD, coronary artery disease; eGFR, estimated glomerular filtration rate; HD, hemodialysis; MDRD, modification of diet in renal disease; n.a., non-applicable.

^a^ Mean ± S.D.;

^b^ n (%).

Systolic function of the left ventricle, as analyzed by the ejection fraction at the echocardiogram, was similar between groups (66.1±10.1% *vs*. 68.6±5.4% for HD patients and controls, respectively, P = 0.167) but diastolic dysfunction was more prevalent in HD patients (77% *vs*. 42%, P = 0.004).

There was no difference between HD and control groups in relation to pre-exercise test parameters (HR = 77±12bpm *vs*. 76±13bpm, p = 0.705; systolic blood pressure = 131±39mmHg *vs*. 130±31mmHg, p = 0.860; and diastolic blood pressure = 82±11mmHg *vs*. 83±9mmHg, p = 0.135).

No complications occurred during ETT in HD patients. Reasons for stopping the exercise were: general exhaustion, 80.5%, exhaustion of the lower limbs muscle, 4.9%, left bundle branch block of high-grade, 2.4%, arrhythmia, 2.4%, and hypertension, 2.4%. Cardiorespiratory fitness parameters were significantly different between groups. Hemodialysis patients exhibited lower MET (6.9±2.0 *vs*. 9.5±2.5, p<0.001) and higher functional aerobic impairment (29.5±12.0 *vs*. 2.8±20.1, p<0.001).

Differences were observed regarding the mean values of HR related parameters in ETT between groups. Hemodialysis patients exhibited lower chronotropic index, reduced HRR at 1^st^, 2^nd^, 3^rd^, 4^th^, and 5^th^ min, higher resting HR/HRR index, and reduced HRV during exercise and recovery (Table 2).

**Table 2:** Mean and standard deviation of heart rate related autonomic dysfunction parameters in 24-h Holter and exercise treadmill test.

	HD patients	Controls	P value
24h-Holter			
SDNN, msec	82.6 ± 27.6	119.1 ± 47.6	<0.001
SDANN, msec	74.3 ± 26.8	113.1 ± 43.2	<0.001
rMSSD, msec	17.4 ± 7.7	27.6 ± 11.1	<0.001
TI, msec	23.3 ± 8.4	34.4 ± 9.3	<0.001
Exercise treadmill test			
Chronotropic index	0.58 ± 0.19	0.89 ± 0.12	<0.001
HRR 1^st^ min, bpm	11.9 ± 9.1	19.4 ± 8.6	<0.001
HRR 2^nd^ min, bpm	21.3 ± 12.3	33.9 ± 10.5	<0.001
HRR 3^rd^ min, bpm	33.8 ± 21.3	53.3 ± 18.0	<0.001
HRR 4^th^ min, bpm	38.3 ± 21.9	58.0 ± 15.7	<0.001
HRR 5^th^ min, bpm	40.8 ± 21.3	60.3 ± 14.4	<0.001
Resting HR/HRR index	0.80 ± 0.19	0.69 ± 0.18	0.008
SDNN exercise, msec	33.7 ± 14.2	50.0 ± 21.8	<0.001
rMSSD exercise, msec	11.0 ± 5.5	15.3 ± 14.7	0.092
SDNN recovery, msec	20.1 ± 9.8	27.3 ± 17.4	0.024
rMSSD recovery, msec	11.5 ± 7.8	15.4 ± 17.3	0.196

HR, Heart rate; HRR, Heart rate variation in recovery; TI, triangular index

**Fig 1:**
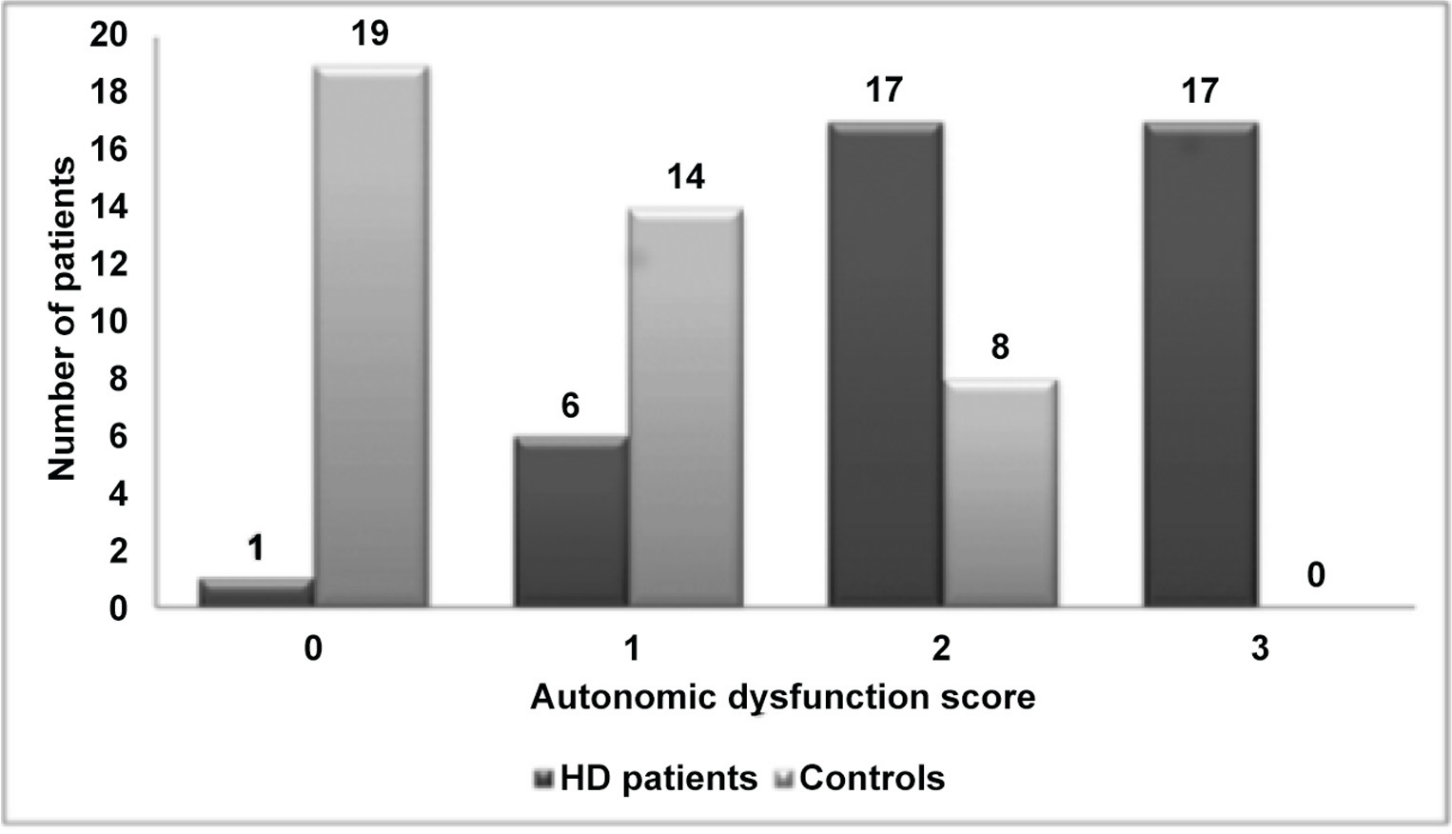
Autonomic dysfunction score. Number of cases with 0, 1, 2, and 3 points in the exercise treadmill test autonomic dysfunction score in controls and hemodialysis patients. Parameters that composed the autonomic dysfunction score were: chronotropic index, heart rate recovery in 1^st^ min and SDNN exercise (one point for each abnormal test). HD, hemodialysis.

In general, the frequency of abnormal results denoting AD tended to be higher in HD patients in both ETT and 24h-Holter (Table 3). The parameters with the best performance to discriminate the two groups at the ETT during exercise (chronotropic index) and recovery (HRR 1^st^min), and the HRV (SDNN exercise) were used to develop an AD score using cutoffs of previous studies [10,15,16]. The number of points in the AD score in controls and HD patients are depicted in Fig 1.

**Table 3:** Frequency of abnormal values in selected parameters of autonomic function at the 24h-Holter and exercise treadmill test.

	HD patients N (%)	Controls N (%)	OR (95% CI)	P value
24h-Holter				
SDNN <50 msec	5 (12.2)	4 (9.8)	-	0.693
SDANN <40 msec	5 (12.2)	3 (7.3)	-	0.433
rMSSD <15 msec	20 (48.8)	3 (7.3)	13.0 (3.4–49.3)	<0.001
TI <15	7 (17.1)	1 (2.4)	8.5 (1–73.0)	0.050
Exercise treadmill test				
Altered chronotropic index ^a^	33 (80.5)	5 (12.2)	29.7 (8.9–100.0)	<0.001
HRR 1^st^ min. bpm <12 bpm	23 (56.1)	3 (7.3)	19.0 (5.0–72.2)	<0.001
HRR 2^nd^ min. bpm <22 bpm	23 (56.1)	5 (12.2)	10.8 (3.5–33.4)	<0.001
Resting HR/HRR Index <0.72	30 (73.2)	22 (53.7)	8.5 (1.0–73.0)	0.050
SDNN exercise <50 msec	34 (82.9)	23 (56.1)	6.*7* (2.0–22.2)	0.002
rMSSD exercise <15 msec	30 (73.2)	31 (75.6)	-	0.724
SDNN recovery <50 msec	38 (92.7)	37 (90.2)	-	0.184
rMSSD recovery <15 msec	32 (78.0)	29 (70.7)	-	0.333

HR, heart rate; HRR, heart rate variation in recovery; TI, triangular index.

^a^ values <0.80 for no betablocker use and <0.62 in use of betablocker. Odds ratio were only showed if P<0.05.

The most discriminative parameter between HD and control groups in ETT (the chronotropic index), and the AD scoring were used as the dependent variables to test associations with the primary interest variable of our study, “hemodialysis”. In every multivariate logistic regression models tested, hemodialysis was independently associated with either altered chronotropic index or AD scoring ≥2. The initial association of HD with the rMSSD in 24h-Holter was no longer present after adjustments (OR = 5.5, P = 0.190, model 5), Table 4.

**Table 4:** Crude and adjusted odds ratios in different multivariate analysis models to test associations of hemodialysis treatment with either abnormal rMSSD at the 24h-Holter, or chronotropic index and autonomic dysfunction score ≥ 2 at the exercise treadmill test.

	rMSSD at 24h-holter	Chronotropic index at ETT	AD score ≥ 2 at ETT
	O.R. (95% C.I.)	O.R. (95% C.I.)	O.R. (95% C.I.)
Crude	13.0 (3.4–49.3) ‡	29.7 (8.9–100,0) ‡	20.0 (6.5–61.5) ‡
Model 1	13.3 (3.5–50.6) ‡	44.9 (10.6–190.0) ‡	26.7 (7.6–94.7) ‡
Model 2	18.8 (3.8–92.7) ‡	80.7 (13.3–492.1) ‡	86.3 (12.5–596.5) ‡
Model 3	19.0 (3.6–100.1) †	134.8 (15.5–1175.1) ‡	235.9 (18.5–3012.8) ‡
Model 4	4.2 (0.6–32.9)	146.2.0 (11.7–1832.5) ‡	687.5 (18.6–25473.2) ‡
Model 5	5.5 (0.4–71.1)	50.1 (3.9–641.6) †	270.9 (7.2–10182.7) †

Models consisted of progressive inclusion of following confounding factors: age, and gender (model 1); smoking, body mass index, and sedentary (model 2); diabetes, clonidine and betablocker use (model 3); hemoglobin, and serum C-reactive protein (model 4); and functional aerobic impairment (model 5). AD, autonomic dysfunction; ETT, exercise treadmill test. *P<0.05,

^†^P<0.01,

^‡^P<0.001.

Finally, in HD patients, C-reactive protein correlated with a number of parameters in ETT, namely, HRR at 1^st^ (r = -0.381, P = 0.017) and 2^nd^ minutes (r = -0.382, P = 0.016), resting HR/HRR index (r = 0.355, P = 0.027), SDNN exercise (r = -0.351, P = 0.033) and the AD score (r = 0.318, P = 0.046). There was no correlation between C-reactive protein and parameters assessing HRV at 24h-Holter.

## Discussion

Autonomic dysfunction is a frequent abnormality in HD patients and a marker of cardiovascular events and death [4,10,17,18]. The ETT, a valuable tool to assess AD, is hardly used in HD patients.

We studied the ability of different autonomic parameters during ETT to diagnose AD in HD patients in comparison to a control group and compared the findings in ETT with the ones of the 24-h Holter.

The functional capacity, as analyzed by MET was lower in HD group, a finding already well documented in HD patients [19,20].

In our sample, body mass index was in the limit of overweight in HD patients and clearly in that range in the control group. This is not unexpected considering that under nutrition was common in HD patients in the past [21] but not in the present and that obesity is a recognized risk factor for cardiovascular diseases [22,23]. The proportion of *diabetes mellitus* in our sample was lower than the ones reported in the international series [24–26], and even lower than the one reported for the Brazilian dialysis population [27] probably as a result of the inclusion and exclusion criteria adopted in the study. Diabetic patients are known to have more disautonomia than no diabetic ones; this fact led us to include diabetes as a confounding factor in the multivariate logistic regression models. In support to the majority of reported series [1,24–26], a substantial number of the HD patients were in use of blood pressure drugs. Consistent with the concept that sympathetic overactivity may play an important role in the hypertension of chronic kidney disease patients, drugs mainly affecting this pathway were more frequently prescribed for HD patients [27]. In addition, serum levels of C-reactive protein were statistically higher in HD patients, who are well recognized as portraying a chronic inflammatory state [28,29].

Consistent with previous reports [30,31], left diastolic dysfunction at the echocardiogram was more frequent in HD patients. In contrast, left ventricular systolic function was similar in both groups perhaps due to our enrollment criteria, which excluded patients with overt heart failure to participate.

Low HRV has been associated with reduced survival in the HD population [3,10,17]. In agreement with previous studies, HRV in the 24-h Holter was lower in the studied HD patients denoting impaired autonomic function [3,4,10,17,32].

When analyzing HR related parameters in ETT, considerable differences were found between groups. We observed higher frequency of chronotropic incompetence in HD patients. An attenuated HR response to exercise has been shown to predict adverse cardiac events in subjects without overt cardiovascular disease [16].Reduced HR response to exercise also predicts major adverse cardiac events among persons with overt or suspected cardiovascular disease even after adjusting for left ventricular function and the severity of exercise-induced myocardial ischemia [33]. In heart failure patients it can be an important cause of exercise intolerance, and in patients not taking beta-blockers, the presence of chronotropic incompetence appears to increase the mortality risk [5,6]. Khan et al [12] followed 3790 middle-aged individuals on beta-blockers referred for exercise testing for 4.5 years and, after adjusting for different variables, found that chronotropic index<0.62 was a strong independent predictor of mortality. The initial exercise-induced increase in HR mainly depends on the vagal withdrawal, whereas further increases in increments depend on the sympathetic activity according to the intensity of exercise [34]. Findings for the present study can be accounted for by the hyperadrenergic state characteristic of HD but also present in heart failure [7], which can lead to compensatory vagal hyperactivity. This in turn could provide inadequate vagal withdrawal during exercise and changes in baroreflex response, hindering the increase in HR in response to exercise [7,35]. In other words, despite the tonic increased sympathetic activity, HD patients have reduced phasic autonomic modulation, a well-recognized risk factor.

In our results HRR was significantly lower in HD group in every minute of recovery. Slow recovery of HR post-exercise reflects an inadequate return of cardiac vagal activity, and has proved to be a good marker of cardiovascular events in both heart disease patients and in healthy individuals [36,37], and also such as a predictor of mortality, independently of the angiographic severity of coronary disease [38]. Reduced HRR had already been reported in chronic kidney disease patients far before renal replacement therapy [39] consistent with the knowledge that the sympathetic hyperactivity starts early in the course of chronic kidney disease [30]. In a study using MIBG scintigraphy in heart failure patients an abnormal HRR was found in patients with high washout rate compared with the normal washout ones again suggesting that adrenergic activation may influence the HRR [40].

SDNN during ETT was significantly lower in HD patients at exercise and recovery. Our findings again may reflect the hyperadrenergic state characteristic of HD patients since SDNN represents a general evaluation of autonomic nervous system balance, which depends on modulation by the sympathetic and parasympathetic branches [10]. Accordingly, the resting HR/HRR index was higher in HD patients than controls. In normal subjects, the resting HR/HRR index, a cardiac autonomic imbalance index, has been directly associated with inflammation and age, and inversely with physical ability [14]. In contrast, the variable predominantly affected by the parasympathetic branch, rMSSD, was not different between groups.

When using cutoffs of greater cardiovascular risk to parameters, we found the ETT was pivotal in identifying dysautonomia in HD patients exhibiting a great ability to discriminate the two groups. In this regard, the higher O.R. values were found for the chronotropic index, and HRR 1^st^min, parameters that are well-recognized as useful tools for the diagnosis of AD at other settings [5,6,11,15].

The presence of more than one abnormal parameter in ETT reinforces the probability of a perturbation in the autonomic system [6]. In a previous study [6], chronotropic incompetence as well as HRR predicts cardiovascular mortality in patients without renal disease referred for exercise testing. Chronotropic incompetence was a stronger predictor of cardiovascular mortality than HRR, but risk was higher if the two abnormalities were simultaneously present. When we applied a score that comprised alterations in three different tests in ETT to our sample, we found that maximal scoring was only observed in HD patients suggesting that these patients may carry a higher risk of mortality.

To better explore the relationship between HD and AD parameters we resort to a variety of models of logistic regression. HD was independently associated with chronotropic incompetence in every tested model including the one in which the functional aerobic impairment was inserted as a controlling variable. Interestingly, the association of hemodialysis with an AD scoring ≥2 was stronger suggesting that this variable portrays the best ability to detect AD in this setting. At this point, it would be convenient to address the issue of over diagnosis of AD by the ETT. The question cannot be definitely answered by our study due to its cross sectional nature, without hard endpoints. However, C-reactive protein levels, which reflect microinflammation, were only associated with parameters measured at the ETT allowing us to think that the findings at the ETT may indeed be clinically relevant. The apparent superiority of the ETT in comparison to the 24h-Holter did not come as a surprise. It is conceivable that the stress faced during exercise imposes substantial requirements to the autonomic system that can surface perturbations that otherwise would remain undetectable.

Reduced number of patients is a limitation of our study that recruited patients from a single center of dialysis. In view of that, our findings need to be confirmed in larger longitudinal studies.

In conclusion, the ETT was feasible and useful to diagnose AD in HD patients. Among the parameters studied, the chronotropic index and the AD score identifying a higher number of affected patients. Findings at ETT correlated with the levels of C-reactive protein suggest that the detected abnormalities may reflect true AD. A larger longitudinal study is necessary to confirm our findings and better evaluate its predictive value.

## References

[1] Lugon JR, Warrak EA, Lugon AS, Salvador BA, Nobrega AC. Revisiting autonomic dysfunction in end-stage renal disease patients. Hemodial Int. 2003;7:198–203. 10.1046/j.1492-7535.2003.00038.x 19379365

[2] Herzog CA, Mangrum JM, Passman R. Sudden cardiac death and dialysis patients. Seminars in Dialysis. 2008; 21:300–7. 10.1111/j.1525-139X.2008.00455.x 18627568

[3] Ranpuria R, Hall M, Chan CT, Unruh M. Renal-heart rate variability (HRV) in kidney failure: measurement and consequences of reduced HRV. Nephrol Dial Transplant. 2008;23:444–9. 18003665 10.1093/ndt/gfm634

[4] Chan CT, Levin NW, Chertow GM, Larive B, Schulman G, Kotanko P, et al Determinants of Cardiac Autonomic dysfunction in ESRD. Clin J Am Soc Nephrol. 2010; 5:1821–7. 10.2215/CJN.03080410 20616163 PMC2974383

[5] Brubaker PH, Kitzman DW. Chronotropic incompetence: causes, consequences, and management. Circulation. 2011;123:1010–20. 10.1161/CIRCULATIONAHA.110.940577 21382903 PMC3065291

[6] Myers J, Tan SY, Abella J, Aleti V, Froelicher V. Comparison of the chronotropic response to exercise and heart rate recovery in predicting CV mortality. Eur J Cardiovasc Prev Rehabil. 2007;14:215–21. 17446799 10.1097/HJR.0b013e328088cb92

[7] Messias LR, Carreira MAMQ, Mesquita CT. Stress Test Evaluation of dysautonomia in heart failure patients. Rev Bras Cardiol. 2010;23:244–50.

[8] Lentine KL, Costa SP, Weir MR, Robb JF, Fleisher LA, Kasiske BL, et al Cardiac disease evaluation and management among kidney and liver transplantation candidates: a scientific statement from the American Heart Association and the American College of Cardiology Foundation. J Am Coll Cardiol. 2012;60:434–80. 10.1016/j.jacc.2012.05.008 22763103

[9] Huang PH, Leu HB, Chen JW, Wu TC, Lu TM, Ding YA, et al Comparison of endothelial vasodilator function inflammatory markers, and N-terminal pro-brain natriuretic peptide in patients with or without chronotropic incompetence to exercise test. Heart. 2006;92:609–14. 16159987 10.1136/hrt.2005.064147 PMC1860951

[10] Fukuta H, Hayamo J, Ishihara S, Sakata S, Mukai S, Ohte N, et al Prognostic value of heart rate variability in patients with end-stage renal disease on chronic haemodialysis. Nephrol Dial Transplant. 2003;18:318–25. 12543887 10.1093/ndt/18.2.318

[11] Meneghelo RS, Araújo CGS, Stein R, Mastrocolla LE, Albuquerque PF, Serra SM, et al III Diretrizes da Sociedade Brasileira de Cardiologia sobre teste ergométrico. Arq Bras Card. 2010;5:1–26. 10.1590/S0066-782X2010000800001 21340292

[12] Khan MN, Pothier CE, Lauer MS. Chronotropic incompetence as a predictor of death among patients with normal electrocardiograms taking beta blockers (metoprolol or atenolol). Am J Cardiol. 2005;96:1328–33. 16253608 10.1016/j.amjcard.2005.06.082

[13] Shetler K, Marcus R, Froelicher VF, Vora S, Kalisetti D, Prakash M, et al Heart rate recovery: validation and methodologic issues. J Am Coll Cardiol. 2001;38:1980–7. 11738304 10.1016/s0735-1097(01)01652-7

[14] Jae SY, Heffernan KS, Yoon ES, Lee MK, Fernhall B, Park WH. The inverse association between cardiorespiratory fitness and C-reactive protein is mediated by autonomic function: a possible role of the cholinergic antiinflammatory pathway. Mol Med. 2009;15:291–6. 10.2119/molmed.2009.00057 19603105 PMC2710293

[15] Maddox TM, Ross C, Ho M, Masoudi FA, Magid D, Daugherty SL, et al The prognostic importance of abnormal heart rate recovery and chronotropic response among exercise treadmill test patients. Am Heart J. 2008;156:736–44. 10.1016/j.ahj.2008.05.025 18926155

[16] Savonen KP, Lakka TA, Laukkanen JA, Rauramaa TH, Salonen TJ, Rauramaa R. Usefulness of chronotropic incompetence in response to exercise as a predictor of myocardial infarction in middle-aged men without cardiovascular disease. Am J Cardiol. 2008;101:992–8. 10.1016/j.amjcard.2007.11.045 18359320

[17] Oikawa K, Ishihara R, Maeda T, Yamaguchi K, Koike A, Kawaguchi H, et al Prognostic value of heart rate variability in patients with renal failure on hemodialysis. Int J Cardiol. 2009;131:370–7. 10.1016/j.ijcard.2007.10.033 18199499

[18] Brotman DJ, Bash LD, Qayyum R, Crews D, Whitsel EA, Astor BC, et al Heart rate variability predicts ESRD and CKD-related hospitalization. J Am Soc Nephrol. 2010;21:1560–70. 10.1681/ASN.2009111112 20616169 PMC3013524

[19] Painter P. Determinants of exercise capacity in CKD patients treated with hemodialysis. Adv Chronic Kidney Dis. 2009;16:437–48. 10.1053/j.ackd.2009.09.002 19801134

[20] Fotbolcu H, Duman D, Ecder SA, Oduncu V, Cevik C, Tigen K, et al Attenuated cardiovascular response to sympathetic system activation during exercise in patients with dialysis-induced hypotension. Am J Nephrol. 2011;33:491–8. 10.1159/000327829 21546765

[21] Stenvinkel P, Heimbürger O, Lindholm B, Kaysen GA, Bergström J. Are there two types of malnutrition in chronic renal failure? Evidence for relationships between malnutrition, inflammation and atherosclerosis (MIA syndrome). Nephrol Dial Transplant. 2000;15:953–60. 10862630 10.1093/ndt/15.7.953

[22] Bonanni A, Mannucci I, Verzola D, Sofia A, Saffioti S, Gianetta E, et al Protein-Energy Wasting and Mortality in Chronic Kidney Disease. Int J Environ Res Public Health. 2011;8:1631–54. 10.3390/ijerph8051631 21655142 PMC3108132

[23] Ikizler TA, Cano NJ, Franch H, Fouque D, Himmelfarb J, Kalantar-Zadeh K, et al Prevention and treatment of protein energy wasting in chronic kidney disease patients: a consensus statement by the International Society of Renal Nutrition and Metabolism. Kidney Int. 2013;84:1096–107. 10.1038/ki.2013.147 23698226

[24] United States Renal Data System. USRDS 2012 Annual data report: atlas of chronic kidney disease & end-stage renal disease in the United States Cardiovascular disease: p. 247–258. Bethesda, MA: National Institutes of Health, National Institute of Diabetes and Digestive and Kidney Diseases; 2012.

[25] Foster MF, Rawlings AM, Marrett E, Neff D, Willis K, Inker LA, et al Cardiovascular risk factor burden, treatment, and control among adults with chronic kidney disease in the United States. Am Heart. J 2013;166:150–6. 10.1016/j.ahj.2013.03.016 23816034 PMC3933201

[26] Sesso RC, Lopes AA, Thomé FS, Lugon JR, Watanabe Y, Santos DR. Chronic dialysis in Brazil: report of the Brazilian dialysis census, 2011. J Bras Nefrol. 2012;34:272–7. 23099833 10.5935/0101-2800.20120009

[27] Joles JA, Koomans HA. Causes and consequences of increased sympathetic activity in renal disease. Hypertension. 2004;43:699–706. 14981063 10.1161/01.HYP.0000121881.77212.b1

[28] Jofré R, Rodriguez-Benitez P, López-Gómez JM, Pérez-Garcia R. Inflammatory syndrome in patients on hemodialysis. J Am Soc Nephrol. 2006;17:S274–S280. 17130274 10.1681/ASN.2006080926

[29] Sharain K, Hoppensteadt D, Bansal V, Singh A, Fareed J. Progressive increase of inflammatory biomarkers in chronic kidney disease and end-stage renal disease. Clin Appl Thromb Hemost. 2013;19:303–30 10.1177/1076029612454935 22865783

[30] Ie EHY, Zietse R. Evaluation of cardiac function in the dialysis patient—a primer for the non-expert. Nephrol Dial Transplant. 2006;21:1474–81. 16611678 10.1093/ndt/gfl167

[31] Jakubovic BD, Wald R, Goldstein MB, Leong-Poi H, Yuen DA, Perl J, et al Comparative assessment of 2-dimensional echocardiography vs cardiac magnetic resonance imaging in measuring left ventricular mass in patients with and without end-stage renal disease. Can J Cardiol. 2013;29:384–90. 10.1016/j.cjca.2012.07.013 23103220

[32] Rubinger R, Revis N, Pollak A, Luria MH, Sapoznikov D. Predictors of haemodynamic instability and heart rate variability during haemodialysis. Nephrol Dial Transplant. 2004;19:2053–60. 15161953 10.1093/ndt/gfh306

[33] Elhendy A, Mahoney DW, Khandheria BK, Burger K, Pellikka PA. Prognostic significance of impairment of heart rate response to exercise impact of left ventricular function and myocardial ischemia. J Am Coll Cardiol. 2003;42:823–30. 12957427 10.1016/s0735-1097(03)00832-5

[34] Nobrega AC, Araujo CG. Heart rate transient at the onset of active and passive dynamic exercise. Med Sci Sports Exerc. 1993;25:37–41. 8423755

[35] Routledge HC, Townend JN. Why does heart rate response to exercise predict adverse cardiac events? Heart. 2006; 92:577–8. 16387820 10.1136/hrt.2005.079400 PMC1860924

[36] Messias LR, Carreira MAMQ, Miranda SMR, Azevedo JC, Gava IA, Rodrigues RC, et al Relationship between cardiac adrenergic image and exercise testing in heart failure. Arq Bras Cardiol. 2011;96:370–6. 21503390 10.1590/s0066-782x2011005000044

[37] Gayda M, Bourassa MG, Tardif JC, Fortier A, Juneau M, Nigam A. Heart rate recovery after exercise and long-term prognosis in patients with coronary artery disease. Can J Cardiol. 2012;28:201–7. 10.1016/j.cjca.2011.12.004 22336522

[38] Vivekananthan DP, Blackstone EH, Pothier CE, Lauer MS. Heart rate recovery after exercise is a predictor of mortality, independent of the angiographic severity of coronary disease. J Am Coll Cardiol. 2003;42:831–8. 12957428 10.1016/s0735-1097(03)00833-7

[39] Késói I, Sági B, Vas T, Kovács T, Wittmann I, Nagy J. Heart rate recovery after exercise is associated with renal function in patients with a homogenous chronic renal disease. Nephrol Dial Transplant. 2010;25:509–13. 10.1093/ndt/gfp504 19783602

[40] Messias LR, Carreira MAMQ, Miranda SMR, Azevedo JC, Gava IA, Rodrigues RC, et al Is abnormal adrenergic activation associated with abnormal heart rate recovery? Arq Bras Cardiol. 2012;98:398–405. 22450335 10.1590/s0066-782x2012005000027

